# Dose-dependent effects of recombinant spike protein-based vaccination on SARS-CoV-2 evolutionary dynamics and the emergence of variants in hamster models

**DOI:** 10.1099/jgv.0.002314

**Published:** 2026-08-19

**Authors:** Kyuyoung Shim, Jeong Hwa Choi, Eun-Ha Hwang, Green Kim, Gyu-Seo Bae, Eunsu Jeon, Seung Ho Baek, Hee-Sung Kim, Seok Hwan Kim, Dae-Soo Kim, Kiwon Jang, Jung Joo Hong, Bon-Sang Koo

**Affiliations:** 1 Korea Research Institute of Bioscience and Biotechnology, Cheongju, Republic of Korea; 2 KRIBB School of Bioscience, Korea University of Science and Technology, Daejeon, Republic of Korea; 3 College of Medicine, Chungbuk National University, Cheongju, Republic of Korea; 4 College of Medicine, Chungnam National University, Daejeon, Republic of Korea

**Keywords:** delta variant, intra-host single-nucleotide variants, Omicron variant, SARS-CoV-2, viral evolution

## Abstract

Severe acute respiratory syndrome coronavirus 2 (SARS-CoV-2) has continued to circulate globally through the persistent emergence of novel variants. Vaccination has been regarded as one of the selective environments that can influence SARS-CoV-2 evolution by exerting immune pressure. This study investigated the evolutionary dynamics of the SARS-CoV-2 Delta variant in hamsters immunized with varying doses of a full-length spike protein vaccine, potentially reflecting the heterogeneous levels of immunity. In hamster models, higher vaccine doses prevented viral replication in the lungs but only partially suppressed replication in the nasal passages. After excluding intra-host single-nucleotide variants (iSNVs) detected in non-vaccinated controls, a negative binomial model adjusting for read depth revealed a significant vaccine dose-dependent increase in iSNV occurrence in the Spike, ORF1a, ORF1b and ORF3a genes in nasal samples and positive selection signals were predominantly observed in the highest vaccine dose group. The iSNVs were observed in diverse and distinct combinations that were unique to each individual, reflecting host-specific intra-host mutation patterns. Some aa substitutions detected in at least two individuals in the vaccinated group were more frequently observed in the Omicron variant. *In silico* analyses incorporating individual-specific iSNVs demonstrated that reduced binding affinity to class 1 and 3 neutralizing antibodies was observed exclusively in variants identified from certain vaccinated individuals. In conclusion, these findings indicate that heterogeneous vaccine-induced immune pressure can shape intra-host SARS-CoV-2 evolution in a dose-dependent and host-specific manner and highlight the potential role of partial immunity and increased iSNVs in the upper respiratory tract in driving the emergence of putative immune-evasive viral variants.

**Impact Statement**

Severe acute respiratory syndrome coronavirus 2 (SARS-CoV-2) continues to circulate worldwide due to the emergence of viral variants, even in populations with immunity from vaccination or prior infection. Certain viral evolutionary trajectories are known to be shaped by vaccine-induced selective pressure. In this study, we modelled this heterogeneity by varying vaccine doses to mimic heterogeneous immune states and examined how viral mutations are shaped under these conditions. Current vaccine platforms have been shown to be less effective at suppressing viral replication in the upper respiratory tract (URT) than in the lower respiratory tract. We found that more frequent viral mutations were observed in the URT as vaccine dose increased. Importantly, vaccine-associated mutational changes were not confined to the spike protein, the primary target of vaccination but were also detected across multiple non-spike viral proteins. In addition, most mutations were unique to individual hosts. These findings enhance our understanding of how different vaccine-induced immune states influence SARS-CoV-2 evolution.

## Data availability

All data generated or analysed during this study are included in this article and its Supplementary Information files. The code used for data processing and statistical analysis is publicly available at https://github.com/porco9/SARS-CoV-2_iSNV_analysis. The raw paired-end sequencing reads generated in this study have been deposited in the NCBI Sequence Read Archive (SRA) under BioProject accession number PRJNA1456970. The individual samples are available under BioSample accession numbers SAMN57437899–SAMN57437930. Individual BioSample and SRA run accessions, together with the corresponding study sample identifiers and R1 and R2 FASTQ filenames, are provided in Supplementary Table S7.

## Introduction

Severe acute respiratory syndrome coronavirus 2 (SARS-CoV-2) infections have continued to occur worldwide due to multiple variants with distinct phenotypic characteristics [1, 2]. The spike protein of SARS-CoV-2 exhibited an evolutionary rate of ∼10⁻³ substitutions per site per year, which is relatively rapid compared to other viruses [3, 4]. Until now, real-world evolutionary characteristics of this virus could be classified into three steps [4, 5]. In the first step, there were no significant mutations during the first 8 months after the first outbreak by Wuhan-Hu-1. After that, several variants of concern (VOCs) were serially emerged and replaced the previous dominant variants, including alpha, beta, gamma and delta variants. These VOCs mainly represented increased transmissibility, virulence and immune escape. In the final stage of SARS-CoV-2 evolution, Omicron variants exhibited markedly distinct phenotypes compared to previous VOCs, including decreased virulence, high transmissibility and increased upper respiratory tract (URT) affinity [5]. To date, Omicron sublineages have continued to emerge and co-circulate, playing a dominant role in SARS-CoV-2 infections.

Most mutations in SARS-CoV-2 are neutral or mildly deleterious, with no significant phenotypic effects on the host [6]. However, some mutations in specific regions, particularly the receptor-binding domain (RBD) and the N-terminal domain (NTD) of the spike protein, can directly alter angiotensin-converting enzyme 2 (ACE2) binding affinity, immune evasion and protein fold stability [2]. Therefore, non-synonymous (NS) mutations in the SARS-CoV-2 spike protein may influence viral virulence, tissue tropism and transmissibility. Among VOCs, the Omicron variants represented over 60 aa mutations on spike protein, including 15 mutations in the RBD compared to the Wuhan-Hu-1 strain, whereas pre-Omicron VOCs, delta variants, have only less than 30 aa motifs [7]. Omicron has faster replication in the URT but less in the lungs due to the shifted entry mechanisms from fusion to endocytosis, causing significantly increased transmissibility and less virulence [7, 8].

Vaccination and natural infection can contribute to SARS-CoV-2 evolution through immune-mediated selective pressure [7]. During the delta wave, boosting vaccination was implemented in populations. Although immune escape has been observed in earlier variants, such as the Beta variant, it has been most prominently reported in the Omicron variant [9]. These Omicron variants escape the existing antibody responses in people boosting vaccination and existing neutralizing antibodies [10, 11]. Interestingly, immune imprinting was observed in Omicron-infected people receiving SARS-CoV-2 vaccination [12]. In a previous report, BA2- and BA5-specific B cells were rarely produced in vaccinated people infected with these Omicron sublineages [12]. This phenomenon could induce convergent virus evolution of Omicron sublineages. Therefore, Omicron variants harbouring multiple mutations can escape existing humoral immune responses in sera from convalescent and vaccinated people [12]. Over 85% of the neutralizing antibodies proved to escape Omicron variants which located in the main six antibody epitopes in SARS-CoV-2 [11]. In real-world settings, SARS-CoV-2 breakthrough infections and reinfections were predominantly associated with Omicron variants.

Although the exact mechanisms underlying the emergence of highly divergent variants such as Omicron remain unclear, several hypotheses have been proposed to explain this phenomenon [6]. First, new variants were already circulating in populations even though this virus was not detectable, especially in low-income countries with very low diagnosis rates [13]. Also, zoonotic origins of new variants were speculated, especially three animal species including Syrian hamster, mink and white-tailed deer [14, 15]. Among them, Omicron BA.1 could effectively infect only Syrian hamsters. Lastly, the origin of this SARS-CoV-2 variant was suspected in immunocompromised patients with chronic SARS-CoV-2 infection. They were repeatedly treated with monoclonal and convalescent plasma and had weak immune responses by vaccination [5, 16, 17]. This vaccination and therapeutics can induce selection pressure on the virus in patients without complete virus clearance for many months. High rates of NS mutations in the spike protein were identified in these patients, although there were no consistent mutation patterns [16, 17].

In this study, we analysed the mutational patterns and computationally predicted phenotypic characteristics of SARS-CoV-2 in Syrian hamsters inoculated with the Delta strain that circulated during the boosting vaccination period. Because human studies are confounded by variability in exposure dose, immune status and timing of infection, we employed a controlled animal model to directly assess how vaccine-induced immune pressure influences viral diversity. To simulate varying levels of immune pressure, animals were vaccinated with different concentrations of the recombinant spike protein, reflecting the heterogeneous immunity. To focus on mutation patterns associated with immune pressure, we excluded variants observed in the unvaccinated group and removed mutations likely attributable to host adaptation, thereby analysing vaccine dose-dependent intra-host single-nucleotide variant (iSNV) patterns. Furthermore, we evaluated the extent to which the observed mutations are present in SARS-CoV-2 sequences isolated from human populations, including their relative frequencies in variants such as Omicron and Delta.

## Methods

### Virus and vaccine

A SARS-CoV-2 Delta variant B.1.617.2 (registration number NCCP 43390), isolated from a Korean patient with SARS-CoV-2, was provided by the National Culture Collection for Pathogens (Cheongju, Korea). The virus was passaged twice in Vero cells cultured in Dulbecco’s Modified Eagle Medium (Welgene Inc.) supplemented with 2% FBS and 1% penicillin (10,000 IU ml^−1^) at 37 °C in a 5% CO₂ incubator. Three days post-inoculation, the cell culture supernatant was harvested by centrifugation at 3,000 ***g*** for 10 min and stored at −80 °C until use. Virus titration was performed in Vero cells and expressed as the 50% tissue culture infectious dose per millilitre (TCID₅₀ ml^−1^), calculated using the Reed–Muench method [18].

### Animal experiments

**Fig. 1.**
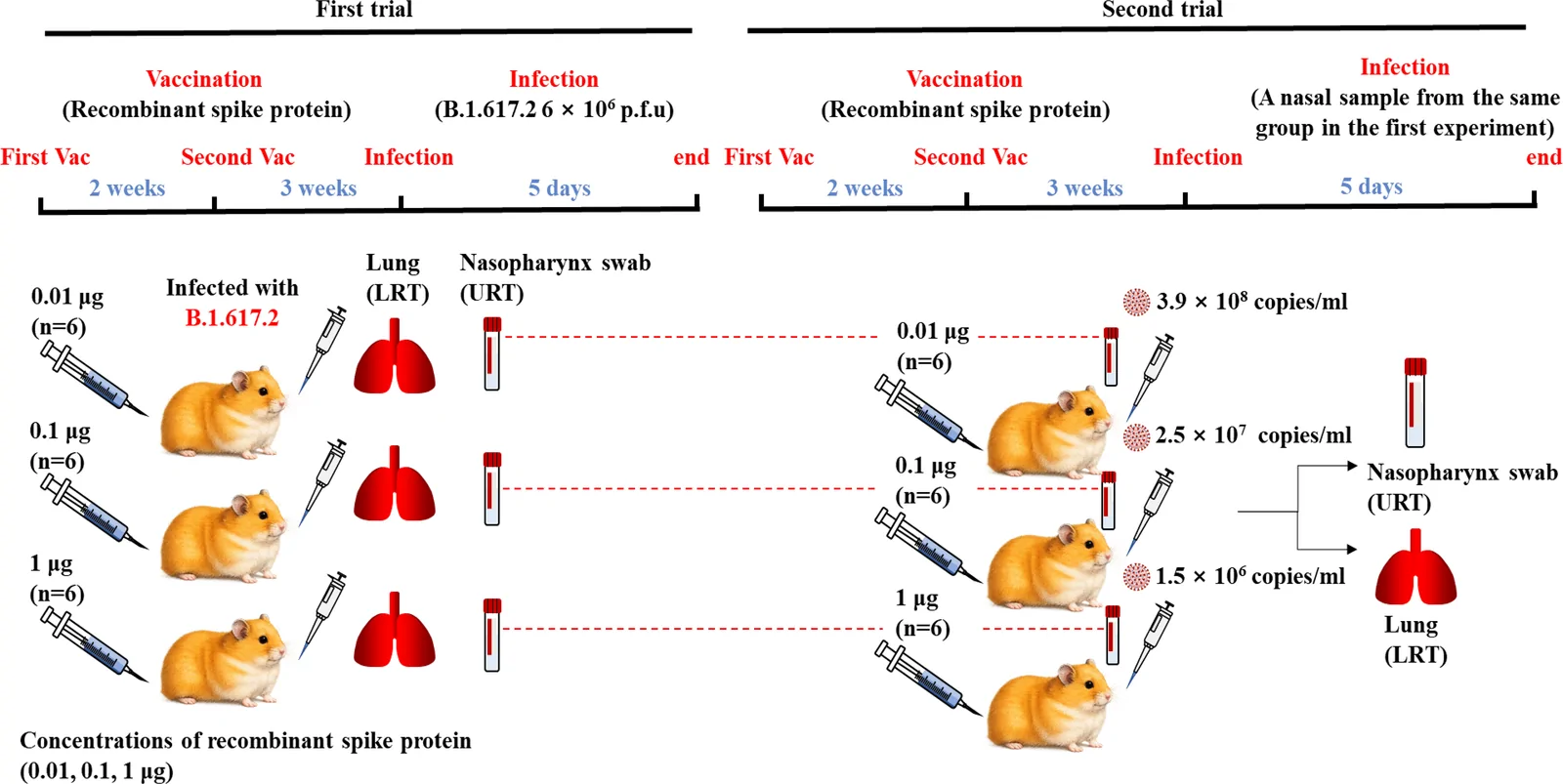
Animal experiment designs and timeline for vaccination and infection.

The animal experiments were conducted twice using identical procedures, with the only difference being the virus used for the challenge inoculation (Fig. 1). Male specific-pathogen-free Golden Syrian hamsters (*Mesocricetus auratus*), aged 4 weeks, were intramuscularly vaccinated with the recombinant spike protein derived from the SARS-CoV-2 Wuhan-Hu-1 strain at doses of 0.01, 0.1 or 1 µg, formulated with 15 µg of Matrix adjuvant (Fig. 1). The vaccination procedure consisted of two doses administered to boost immune responses, with the second dose given 2 weeks after the initial immunization. Three weeks after the second vaccination, the animals were intranasally challenged with the SARS-CoV-2 Delta variant (lineage B.1.617.2), with 40 µl administered into each nostril (80 µl total). In the first trial, a dose of 6×10⁶ p.f.u. derived from cell culture supernatant was administered. In the second trial, a dose ranging from 1.5×10^6^ to 3.9×10^8^ virus copies ml^−1^ obtained from a nasal sample was used for challenge (Fig. 1), in which infectious virus was not detectable by plaque assay (p.f.u. ≈ 0), despite the presence of viral RNA. This sample was selected from the individual showing the highest viral load within the same vaccine dose group in the first trial. Notably, for both the first and second trials, the positive control group was consistently inoculated with 6×10⁶ p.f.u. All challenges were performed under anaesthesia induced with ketamine hydrochloride (100 mg kg^−1^) and xylazine (5 mg kg^−1^). Body weight and clinical signs were monitored daily. At 5 days post-infection (dpi), necropsy was performed, and gross lesions were examined according to standard necropsy procedures. Nasal swabs and left lung tissues were aseptically collected for viral quantification, expressed as viral copy number ml^−1^. Right lung tissues were fixed in formalin and processed for haemotoxylin and eosin staining. Histological scoring of lung sections was conducted based on the established criteria (Table S1). All procedures were approved by the Institutional Animal Care and Use Committee in KRIBB (IACUC Approval No. KRIBB-AEC-22294).

### Viral quantification and pro-inflammatory profiling

Viral RNA was extracted from lung tissues and nasal swab diluents collected from hamsters at 5 dpi. Lung tissues were mechanically disrupted and homogenized in 1 ml of TRIzol reagent (Invitrogen). Nasopharyngeal swab samples were initially diluted in 1 ml of PBS vortexed thoroughly, briefly centrifuged and the supernatant was filtered through a 0.22 µm syringe filter into 2 ml of PBS. The filtered solution was then mixed with 1 ml of TRIzol reagent for RNA extraction. RNA extraction was performed according to the manufacturer’s protocol and all RNA samples were stored at −70 °C until further analysis. Quantitative reverse transcription polymerase chain reaction (qRT-PCR) was performed on all samples, including RNA-positive and -negative controls, using a primer set targeting a partial region of the ORF 1b gene [19]. The expression levels of proinflammatory cytokines including interferon gamma (IFN-γ), interleukin-10 (IL-10), interleukin-6 (IL-6), tumour necrosis factor alpha (TNF-α), macrophage inflammatory protein 1 (MIP-1) and interferon gamma-induced protein 10 (IP-10) were assessed in lung tissue samples using SYBR Green-based real-time PCR. Gene expression was normalized to β-actin, which served as the housekeeping gene [20, 21].

### SARS-CoV-2 library preparation, quality control and whole-genome sequencing

Whole-genome sequencing was performed on samples that tested positive for SARS-CoV-2 by qRT-PCR. The sequencing library was prepared through a three-step process comprising cDNA synthesis, pooling and indexing, using the QIAseq DIRECT SARS-CoV-2 Kit, QIAseq DIRECT SARS-CoV-2 Enhancer and QIAseq SARS-CoV-2 Region Booster (Qiagen). For cDNA synthesis, reverse transcription PCR was performed on viral RNA templates using random hexamer primers. Subsequently, PCR amplification of the cDNA was carried out using multiplex primer pools, including DIRECT SARS-CoV-2 Pool 1 and Pool 2, as well as DIRECT Booster A Pool 1 and Pool 2. After amplification, the contents of all pools for each sample were combined into a single tube and purified using the QIAseq 2X Bead Cleanup kit (Qiagen), following the manufacturer’s instructions. Library amplification and indexing were then performed using the QIAseq DIRECT UDI Index Set B. Following the clean-up procedure, the quality of the sequencing libraries was assessed using a Qubit 4 Fluorometer with the Qubit dsDNA HS Assay Kit (Invitrogen). For sequencing, the SARS-CoV-2 libraries were denatured with 0.2 N NaOH, diluted to a final concentration of 10 pM in HT1 buffer and loaded onto an Illumina MiSeq instrument using the MiSeq V2 kit (300 cycles).

### Intrahost variation profiling

Paired-end sequence data in a FASTQ format were imported and processed using CLC Genomics Workbench 23.0.4 (QIAGEN). During the trimming step, reads with a quality score below 0.01, any ambiguous bases, lengths shorter than 64 nts or adapter sequences were removed. The trimmed reads were then mapped to the Wuhan-Hu-1 reference genome (GenBank accession number MN908947) using the following parameters: match score=1, mismatch cost=2, insertion cost=3, deletion cost=3, length fraction=0.5 and similarity fraction=0.8. The iSNVs were identified based on regions with at least 93% genome coverage and the presence of minor alleles with a frequency greater than 5%, provided the total read depth at the position exceeded 100, using the Wuhan-Hu-1 genome as reference. Importantly, all read depth and minor allele frequency thresholds were applied independently to each individual sample, and pooled reads across samples were not used for variant detection. This conservative threshold ensured that each identified iSNV was supported by a minimum of five independent reads. Aa changes were inferred either when specific iSNVs were identified and the observed minor alleles corresponded to aa substitutions or when previously identified iSNV positions showed altered relative frequencies between major and minor alleles. To exclude iSNVs potentially associated with hamster or cell culture adaptation, analyses of mutations related to vaccine-induced selection pressure were performed after removing variants detected in the positive control group. Aa changes associated with specific anatomical sites were inferred from iSNVs that were exclusively observed either in nasal swabs or in lung samples. To account for differences in sequencing coverage and mutational opportunities, all analyses were conducted using callable site-based normalization (genomic positions with read depth ≥100×).

### Negative binomial regression analysis in iSNVs

For each gene, iSNV counts were modelled as a function of vaccine dose using negative binomial (NB) regression, with log_₁₀_-transformed dose as the predictor. Total iSNV counts were fit in a separate gene-level model, whereas the NS and synonymous incidence rate ratios (IRRs) were obtained from the interaction model described below. Each model included a fixed offset equal to the natural log of the callable fraction, defined as the number of callable sites divided by the gene reference length. For gene *g*, the per-sample count ***y*** was modelled as *y*~NB(μ, θ) with log(μ) = β₀ + **β₁** log₁₀(dose) +log(callable_*g* **/ L_***g*), where callable_*g* and L**_***g* are the callable sites and reference length of gene ***g***, and the offset term adjusts for differences in callable coverage across samples so that β₁ describes the change in iSNV density rather than raw count. Possible-site counts were used only for the *pN/pS* analysis and were not included in the offset. Models were fit on dosed samples only, with the positive-control group excluded. The IRR per 10-fold increase in dose was obtained as exp(β₁), with a 95% confidence interval of exp(β₁±1.96 SE(β₁))**,** using the dose term β₁ for total and NS iSNVs, and the dose term plus the class-by-dose interaction term for synonymous iSNVs. To test whether the dose response differed between NS and synonymous iSNVs, an interaction model combining mutation class (NS as the reference) and log_₁₀_ dose was fit with the same offset, and the ratio of the synonymous to the NS IRR was obtained by exponentiating the interaction coefficient.

### Selection pressure analysis in iSNVs

To estimate selection pressure under vaccination, we quantified the relative enrichment of NS versus synonymous iSNVs across viral genes using an iSNV-based framework [22]. iSNVs were classified as synonymous or NS based on whether the substitution from the major allele to the minor allele, relative to the inoculated virus sequence, resulted in an aa change. Possible synonymous and NS sites were enumerated per gene following the Nei–Gojobori convention. For gene *g* in sample *i*, the NS rate was **pN**_*g,i*=N_*g,i* / Pn_*g***,** where N_*g,i* is the observed number of NS iSNVs and Pn_*g* the number of possible NS sites for that gene. Because synonymous iSNVs were infrequently detected at the gene level, the synonymous rate was estimated once per sample as a genome-wide value, **pS**_***i*** = (Σ_*g* S_*g,i*) / (Σ_*g* **Ps_***g*)**,** where S_*g,i* and Ps_*g* are the observed synonymous iSNVs and possible synonymous sites and the sums run over the analysed genes excluding ORF8 and ORF10, which showed atypical synonymous mutation patterns. This single sample-level value (*pS*_*i*) was then used as the common denominator for every gene in that sample, giving ((**pN/pS**){*g,i*} = *pN*{*g,i*} /*pS*_*i*). The values shown in Fig. 4b are the mean±SD of these ratios across samples within each dose group. Within each dataset, differences across dose groups were assessed by the Kruskal–Wallis test and differences between the filtered and unfiltered datasets were assessed for each gene and dose group by the paired Wilcoxon signed-rank test, with samples matched by ID. Analyses were performed on two parallel datasets: filtered iSNVs, in which variants detected in the positive-control group were excluded and unfiltered iSNVs.

### Real-world aa changes frequency

Real-world frequency of specific aa changes related to vaccination and anatomical location was determined based on the Global Initiative on Sharing All Influenza Data (GISAID) database [23]. Sequences were filtered to include only complete genomes, and those with low coverage were excluded. As of March 2026, each aa substitution was queried by inputting the corresponding protein-level mutation into the GISAID analysis platform, from which the total number of sequences harbouring the substitution was obtained. The relative prevalence of each mutation was calculated as the proportion of sequences containing the substitution within major VOCs, including Delta (GISAID clade GK, PANGO lineage B.1.617.2 and AY.) and Omicron (GISAID clade GRA, PANGO lineage BA.).

### *In silico* immune escape prediction

Immune escape potential of each iSNV was determined using EVEscape, based on the predicted impact of individual aa substitutions. The combined effects of multiple aa changes observed in each individual were assessed using High Ambiguity Driven protein-protein DOCKing (HADDOCK) 2.4. EVEscape scores corresponding to each aa substitution found in the SARS-CoV-2 Delta variant (B.1.617.2) were retrieved from the EVEscape webserver [24–26]. For HADDOCK analysis, predictive spike protein tertiary structures were generated using AlphaFold 3 based on the full-length spike protein aa sequences incorporating minor allele variants identified in each individual [25]. Docking evaluations between SARS-CoV-2 spike structure and either ACE2 protein or neutralizing antibodies were performed using HADDOCK 2.4 [27]. The cryo-EM structure of the SARS-CoV-2 Delta variant (B.1.617.2) in complex with the human ACE2 protein was obtained from the Protein Data Bank (PDB ID: 7TEW). Additionally, cryo-EM structures of SARS-CoV-2 proteins in complex with neutralizing antibodies were retrieved from PDB entries 7C01, 7 KMG and 6XDG. Antigen and antibody residues within 5 Å of each other were identified using PyMOL 3.1 and subsequently designated as active residues for HADDOCK docking [28]. The ACE2 and antibody components were extracted from each complex structure using PyMOL 3.1 and used for docking simulations against predicted tertiary structures of spike proteins generated by AlphaFold 3.

### Statistical analysis

Statistical significance of differences in viral kinetics, virulence, cytokine levels and iSNV frequencies among groups was determined using the Mann–Whitney U test. Statistical significance of associations between vaccine dose and iSNV counts in the NB regression models was assessed using Wald tests for the log_₁₀_(dose) coefficient. Differences in *pN*/*pS* across dose groups were assessed using the Kruskal–Wallis test, and pairwise comparisons between the two datasets were conducted using the Wilcoxon signed-rank test. Differences in HADDOCK scores among groups were assessed using the Kruskal–Wallis test; no statistically significant differences were observed (*P*>0.05). Mann–Whitney U test was performed using GraphPad Prism software, version 8.4.3 (GraphPad Software). Other analyses were performed in R (version 4.4.2; R Foundation for Statistical Computing), and visualizations were generated using the ggplot2 and patchwork packages. A *P*-value of less than 0.05 was considered statistically significant.

## Results

### Pathogenicity, viral replication and proinflammatory cytokines

**Fig. 2.**
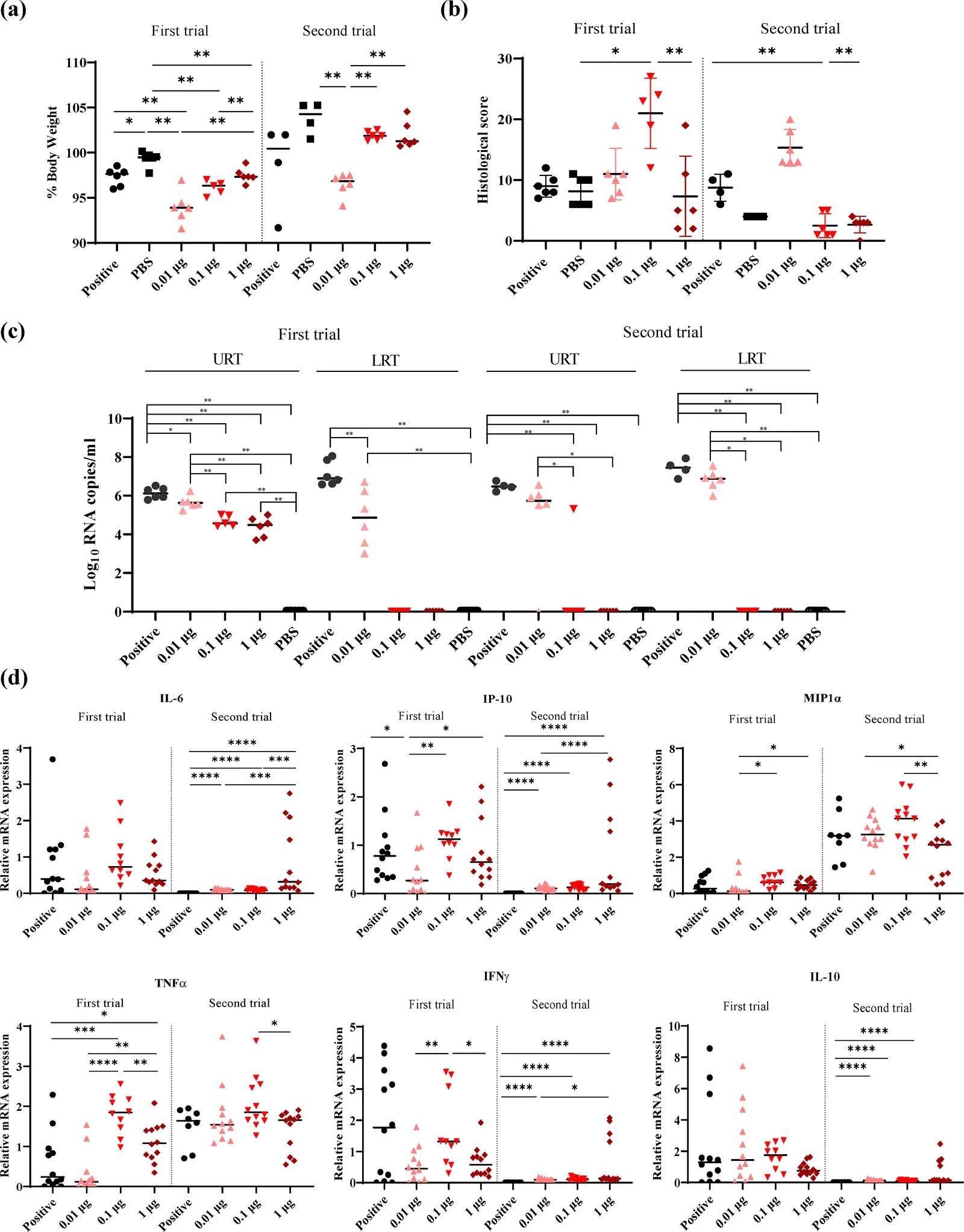
Viral replication, pathogenicity and proinflammatory responses in SARS-CoV-2-infected Syrian hamsters. (**a**) Daily changes in body weight were monitored in hamsters following viral challenge during the first and second trials. Body weight is expressed as a percentage relative to the initial weight at the time of challenge. (**b**) Histopathological scores of lung tissues collected at necropsy on 5 dpi, reflecting the severity of pulmonary lesions. (**c**) Viral RNA levels in samples from the URT and lower respiratory tract (LRT) were quantified at 5 dpi and expressed as log_₁₀_ RNA copies per millilitre. (**d**) Relative mRNA expression levels of proinflammatory cytokines and chemokines (IL-6, IP-10, MIP-1α, TNF-α, IFN-γ and IL-10) were measured in lung tissues at 5 dpi. The positive control group consisted of unvaccinated animals that were challenged with SARS-CoV-2, while the PBS control group consisted of animals that received neither vaccination nor SARS-CoV-2 challenge. Each symbol represents an individual animal, and horizontal bars indicate group means. Statistical significance between groups is indicated (*P<0.05, **P<0.01, ***P<0.001, ****P<0.0001).

Pathogenicity was determined by criteria on body weight changes and histological scoring for lung samples (Fig. 2a, b and Table S1). In the first trial, weight loss was significantly more pronounced in groups receiving lower vaccine doses, demonstrating an inverse dose-dependent relationship between vaccine concentration and body weight reduction (Fig. 2a). Histological scoring revealed the highest scores in the 0.1 µg group (Fig. 2b), a pattern that was not fully consistent with the dose-dependent trend observed for body weight loss. In the secondary trial, significantly greater weight loss was observed in the 0.01 µg group compared with the higher-dose vaccination groups (Fig. 2a), accompanied by markedly elevated histological scores only in this group, indicating consistently increased virulence (Fig. 2b). In both the first and second animal experiments, an inverse relationship was observed between vaccine dose and viral load in both the nasal and lung samples (Fig. 2c). In the first trial, viral RNA in lung samples was detected only in the positive control and 0.01 µg groups, whereas in nasal samples, viral RNA was clearly detected across all groups (Fig. 2c). In the proinflammatory cytokine analysis, increased cytokine levels were predominantly observed in the 0.1 µg group in the primary experiment, whereas in the secondary experiment, cytokine elevation was mainly detected in the 1 µg group (Fig. 2d).

### iSNVs analysis

**Fig. 3.**
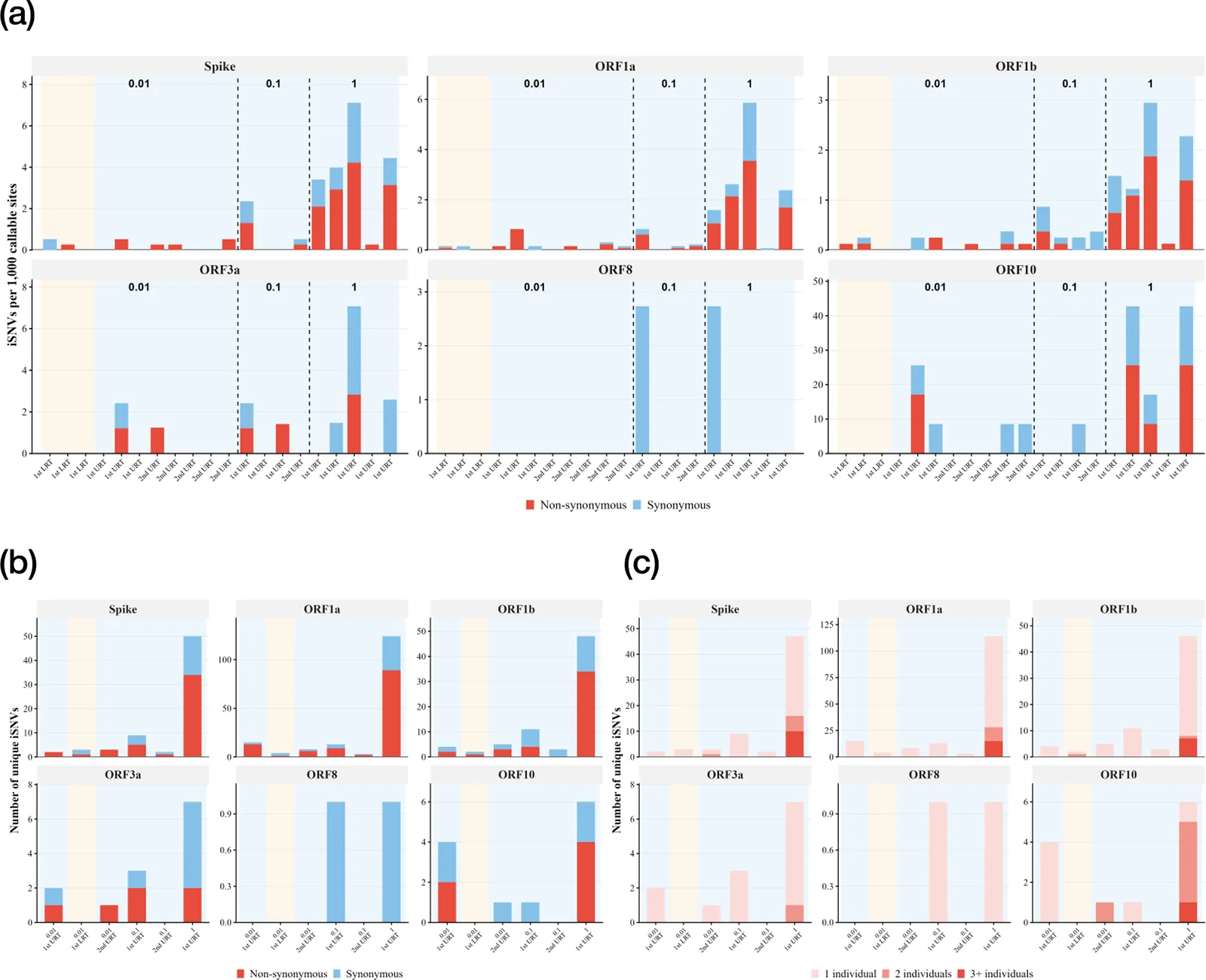
Vaccine dose-dependent gene-specific iSNV density and inter-individual sharing, stratified by mutation type, excluding iSNVs detected in positive control groups. iSNVs were classified as NS and synonymous mutations. iSNV frequency was quantified as the number of iSNVs per 1,000 normalized callable sites for each viral gene across vaccine dose groups. (**a**) Gene-specific iSNV density at the individual level, showing both NS (red) and synonymous (blue) iSNVs across samples. (**b**) Gene-specific iSNV counts at the group level, presented as the number of unique iSNVs per vaccine dose group, separated by mutation type. (**c**) Inter-individual sharing of iSNVs, categorized by the number of animals in which each iSNV was detected (one, two or three or more individuals). Background shading denotes tissue origin, with light blue representing the URT and light orange representing the LRT. Mutation types are indicated by colour (red, NS; blue, synonymous). Vaccine dose groups correspond to 0.01, 0.1 and 1 µg of recombinant spike protein.

In the present study, iSNVs observed in the vaccinated groups were analysed both with and without excluding variants detected in the positive control group. All viral genes were included in the analysis, and the figures display only those genes in which iSNVs were detected. In four ORF3a samples and one ORF1b sample, genome coverage based on a≥100 read depth threshold ranged between 80 and 90%, whereas all other individuals achieved over 93% coverage across all genes and the whole genome. iSNVs per 1,000 normalized callable sites were successfully assessed across viral proteins and vaccination doses (Figs 3a, b and S1). In the unvaccinated positive control group, six animals were analysed, yielding six iSNVs from nasal samples and two from lung samples that met the predefined quality thresholds (Table S2). In the vaccinated group, 17 animals were included, with 17 iSNVs detected in URT samples and 3 in lower respiratory tract (LRT) samples under the same criteria, including sufficient genome coverage and read depth (Table S3). All lung-derived iSNVs were detected in the 0.01 µg dose group in the first trial, whereas nasal iSNVs identified across both the primary and secondary experiments were observed in 8, 4 and 5 animals in the 0.01, 0.1 and 1 µg groups, respectively. These iSNVs were not confined to the spike protein, the primary target of the vaccine but were distributed across multiple non-spike viral proteins (Fig. 3a and b). iSNVs were counted per individual, such that identical variants observed in multiple individuals were counted separately for each individual. Substantial inter-individual variation in iSNV distribution was observed within each group; most iSNVs were unique to individual animals, and the number of these unique iSNVs increased with higher vaccine doses in the first experiment (Fig. 3c).

### NB regression and selection pressure

**Fig. 4.**
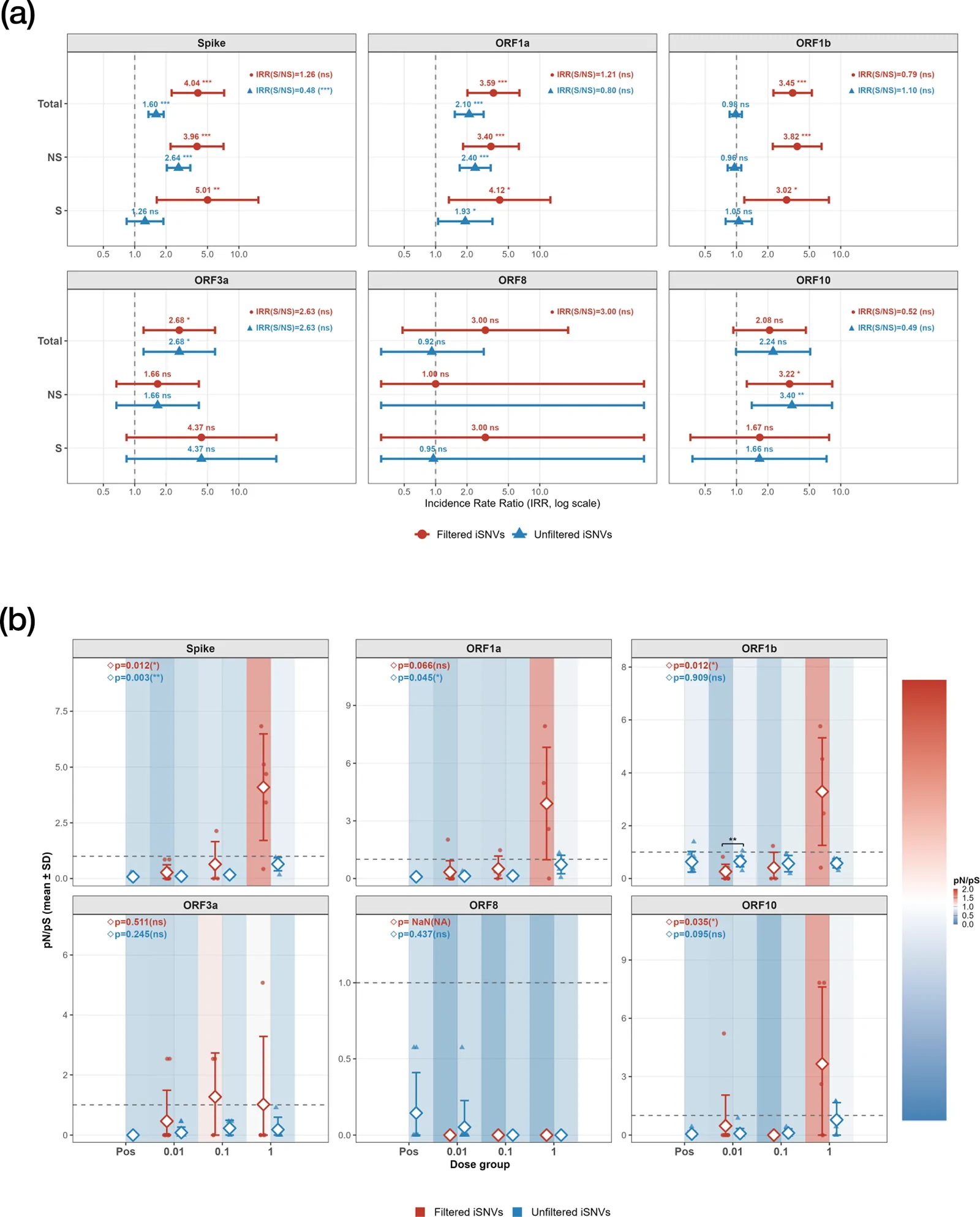
Read-depth-adjusted NB modelling and gene-specific selection pressure analyses of iSNVs. (**a**) Associations between vaccine dose and iSNV counts were evaluated using NB regression models with read-depth-adjusted offsets accounting for callable sites and mutational opportunity. IRRs and 95% confidence intervals are shown for synonymous (**s**), NS and total iSNVs across viral genes. An interaction model (dose×mutation type) was additionally applied to assess differential dose-dependent effects between synonymous and NS mutations, and IRR ratios (S/NS) are indicated. The dashed vertical line denotes an IRR of 1. (**b**) Gene-specific selection pressure was evaluated using *pN*/*pS* ratios, calculated based on NS and synonymous iSNVs. To ensure robust estimation, genome-wide synonymous mutation rates were used as a normalization factor (excluding ORF6, ORF8 and ORF10), particularly for samples with zero synonymous iSNVs. Points represent individual samples, and mean±sd values are indicated. Background shading reflects relative *pN/pS* values, with values <1 indicating purifying selection and values >1 indicating diversifying selection. Statistical significance is indicated as follows: ns, not significant; *P<0.05, **P<0.01 and ***P<0.001. Vaccine dose groups correspond to 0.01, 0.1 and 1 µg of recombinant spike protein. Filtered analyses (red) exclude iSNVs detected in the positive control group, whereas unfiltered analyses (blue) include all detected iSNVs.

NB regression was successfully applied to both filtered and unfiltered datasets, accounting for differences in iSNV detection by incorporating the log of gene-length – normalized callable sites as an offset term. This analysis in filtered dataset revealed a significant association between vaccine dose and increased iSNVs in the Spike (IRR=4.04, *P*<0.001), ORF1a (IRR=3.59, *P*<0.001), ORF1b (IRR=3.45, *P*<0.01) and ORF3a (IRR=2.68, *P*<0.05) (Fig. 4a). A trend of increased selection pressure across multiple viral genes was observed with increasing vaccination dose (Fig. 4b). In the *pN/pS* analysis, the unfiltered dataset showed significant dose-group-dependent differences in selection pressure in the Spike and ORF1a genes, whereas the filtered dataset showed a statistically significant increase in selection pressure in the Spike, ORF1b and ORF10 genes, particularly in the highest vaccine dose group (1 µg). The higher *pN/pS* observed in the filtered dataset was largely driven by the removal of synonymous iSNVs shared with the positive-control hamsters, which reduced the genome-wide *pS* denominator under the assumption that these shared variants represented host-adaptive or background variants.

### Real-world aa change frequency

**Fig. 5.**
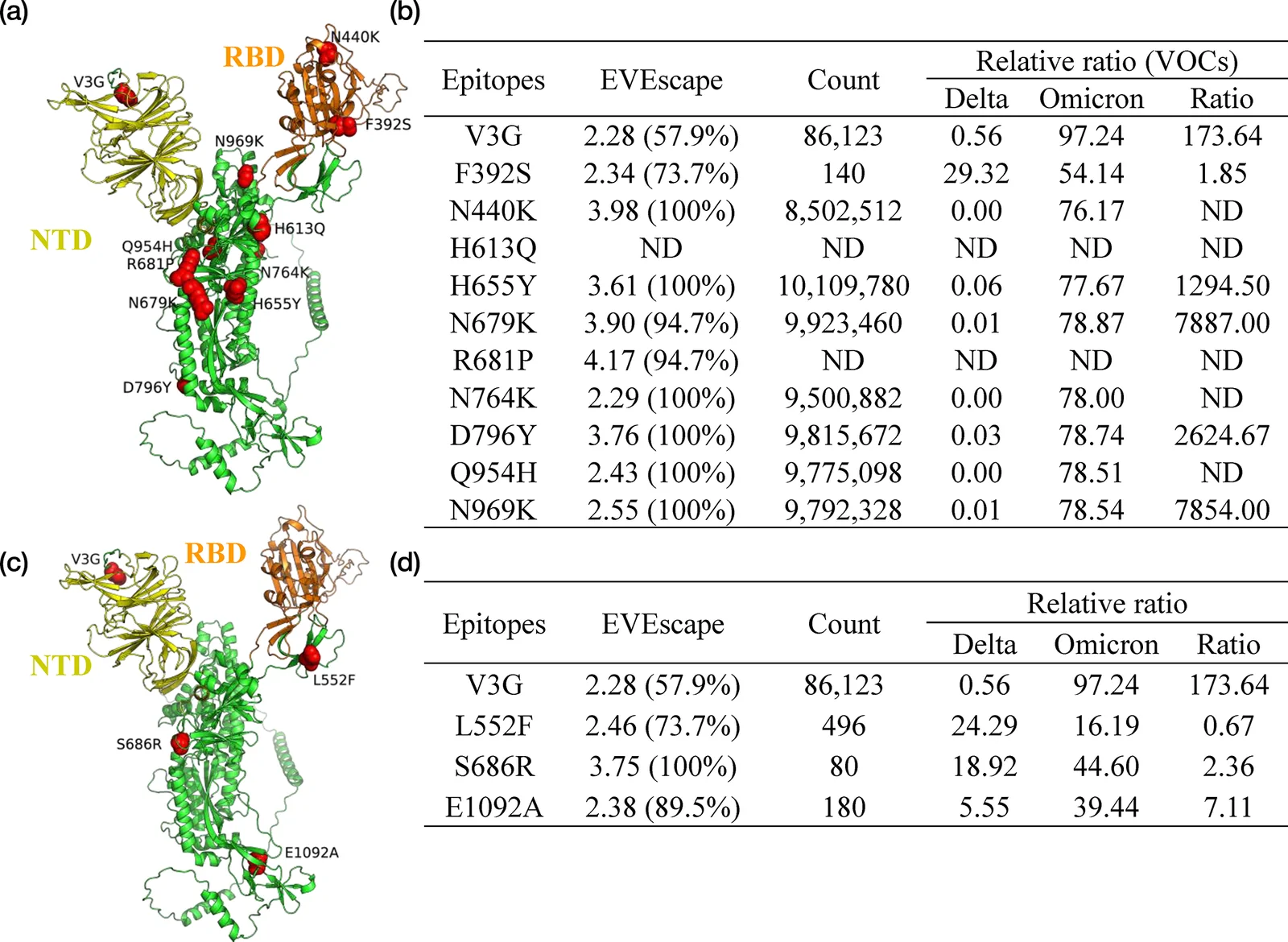
Structural mapping and global prevalence of spike iSNVs with predicted antibody escape properties. (**a**) Spike protein iSNVs identified in more than two vaccinated individuals are shown as red spheres mapped onto the predicted tertiary structure of the SARS-CoV-2 spike protein. (**b**) For iSNVs shown in (**a**), EVEscape scores, corresponding site-specific percentile ranks, global occurrence counts and variant-specific relative frequencies of each iSNV in Delta and Omicron are shown. (**c**) Spike iSNVs identified exclusively in the secondary vaccinated experiment are mapped onto the predicted tertiary structure of the SARS-CoV-2 spike protein. (**d**) EVescape scores, corresponding site-specific percentile ranks, global occurrence counts and variant-specific relative frequencies of the iSNVs shown in (**c**) in Delta and Omicron are shown. Predicted spike protein structures were generated using AlphaFold3 and visualized in PyMOL v3.1. Global occurrence counts were obtained from GISAID (April 2025), and EVescape scores were retrieved from the EVescape webserver. iSNVs detected in the positive control group were excluded. Relative ratio is defined as the frequency of each aa mutation in Omicron divided by that in Delta.

A total of ten iSNVs (V3G, F392S, N440K, H613Q, N679K, R681P, N764K, D796Y, Q954H and N969K) were observed in two or more individuals within the vaccinated groups (Fig. 5). Notably, most of these iSNVs correspond to aa substitutions previously identified in SARS-CoV-2 variants isolated from human patients, and some of these substitutions were observed at higher frequencies in the Omicron variant (Fig. 5a, b). Among the ten iSNVs identified, one was located in NTD and two iSNVs were located in the RBD region. A total of four iSNVs (V3G, L552F, S686R and E1092A) were identified exclusively in the vaccinated groups of the second experiment (Fig. 5c, d). The immune evasion potential of each individual iSNV was assessed using EVEscape scores and their corresponding site-specific percentile ranks (Fig. 5b, d). The iSNVs identified in individual animals from the 0.01, 0.1 and 1 µg vaccination groups in the first experiment are described in Tables S4–S6.

### *In silico* immune escape prediction

**Fig. 6.**
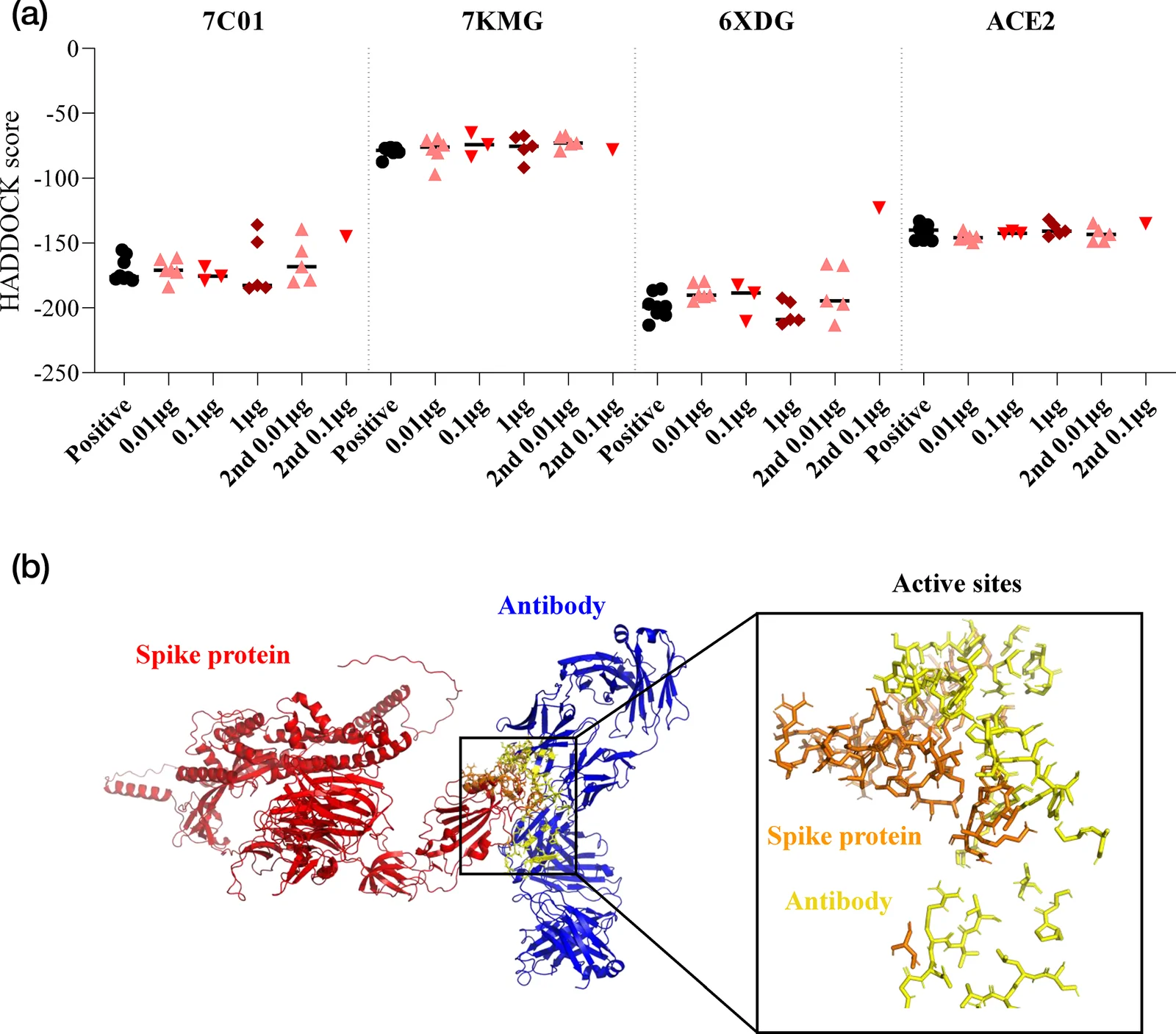
The predicted binding affinity of spike iSNVs for each individual based on *in silico* analysis. (**a**) HADDOCK scores for binding between the SARS-CoV-2 spike protein harbouring iSNVs and three neutralizing antibodies (PDB IDs: 7C01, 7KMG and 6XDG), as well as the human ACE2 receptor (PDB ID: 7TEW), across the first and second experiments. (**d**) Predicted interface between the SARS-CoV-2 spike protein and a neutralizing antibody. Assessment of the combined effects of multiple aa changes observed in each individual using High Ambiguity Driven protein–protein DOCKing (HADDOCK) 2.4. Extraction of ACE2 and antibody components from each complex structure using PyMOL 3.1, followed by docking simulations against predicted tertiary structures of spike proteins generated by AlphaFold 3.

Using AlphaFold 3, spike proteins containing all individual-specific iSNVs were structurally modelled, and their binding affinities with the ACE2 receptor and three major SARS-CoV-2 neutralizing antibodies were successfully assessed using HADDOCK 2.4 (Fig. 6a–c) [27]. *In silico* analyses were performed to evaluate binding affinities between representative class 1–3 SARS-CoV-2 neutralizing antibodies (PDB IDs: 7C01, 7 KMG and 6XDG) and individual-specific viral variants. Among these, the class 2 neutralizing antibody (7 KMG) exhibited lower binding affinity compared with the other neutralizing antibodies in both the first (Fig. 6a). Reduced binding affinity of the class 1 and class 3 neutralizing antibodies (PDB ID: 7C01 and 6XDG) was observed in a subset of individuals from the 1 µg group in the first trial, as well as in some individuals from the vaccinated groups in the second trial (Fig. 6a), indicating inter-individual variability. HADDOCK scores did not differ significantly among groups (Kruskal–Wallis test, *P*>0.05). No consistent changes in HADDOCK-predicted binding affinity between spike proteins and the ACE2 receptor were observed in association with vaccination-induced variation (Fig. 6a). Based on the predicted tertiary structures, active binding sites were defined using a 5 Å distance cutoff, and binding affinities between the SARS-CoV-2 spike protein and neutralizing antibodies were subsequently evaluated (Fig. 6b). Active residues in the SARS-CoV-2 spike protein included positions 343, 401, 403–407, 413–415, 418, 419, 442–445, 447, 448, 450, 451, 453–458, 471–475, 482, 484, 485, 487, 491–493, 498–500, 502 and 503. Active residues in the antibody heavy chain included positions 2, 26–28, 30–33, 52–56, 58, 97, 99–102, 104, 107 and 108, whereas active residues in the antibody light chains included positions 7, 8, 10–13, 15–20, 22, 65–67 and 109 for light chain C and positions 28, 30, 32 and 92–95 for light chain F.

## Discussion

Recently, Omicron sublineages have become globally dominant among SARS-CoV-2 variants, exhibiting distinct characteristics from earlier VOCs [5]. This Omicron variant is hypothesized to have emerged through prolonged SARS-CoV-2 infection in immunocompromised individuals with impaired immunity, as suggested by previous studies [16, 17]. This study assessed the pathogenicity, viral replication and evolutionary dynamics of the SARS-CoV-2 Delta variant in hamster models immunized with varying doses of the recombinant full-length S protein, reflecting heterogeneous immune pressure.

In the first experiment, viral RNA was detected in lung tissues only in the 0.01 µg vaccination group, whereas it was not observed in the 0.1 and 1 µg groups. In contrast, viral RNA was detected in nasal samples across all vaccination groups (0.01, 0.1 and 1 µg), with an inverse correlation between viral load and vaccine dose (Fig. 2C). These findings are related to the limitations of current SARS-CoV-2 vaccines in inducing effective URT mucosal immunity [29, 30]. The viral tissue compartmentalization between the URT and LRT is likely driven by the limited preventive efficacy of vaccination in the URT [31]. Increased viral replication in the lungs is closely associated with elevated pathogenicity, whereas viral loads in the URT are more strongly linked to transmission potential [31, 32]. Consistent with these findings, the present study demonstrated that pathogenicity generally decreased with increasing vaccine dose across both experiments, accompanied by reduced viral replication in the lungs (Fig. 2a and b) [32, 33]. Therefore, current SARS-CoV-2 vaccine platforms demonstrate strong efficacy in mitigating disease severity, but their ability to prevent viral transmission appears to be limited.

Despite vaccination, iSNVs were predominantly detected in viruses identified from nasal samples, and some individuals in the higher-dose vaccination groups tended to harbour a greater number of iSNVs (Fig. 3a). Even within the same vaccine dose group (0.01 µg), a greater number of iSNVs were observed in viruses isolated from nasal tissues than in those from lung tissues (Fig. 3). This dose-responsive trend was not limited to the spike protein, the primary target of the vaccine but was also frequently observed across other viral proteins, including ORF1a, ORF1b and ORF3a, suggesting that vaccine-induced pressure may influence broader regions of the viral genome (Fig. 3). The majority of the iSNVs observed in viral proteins were restricted to a single individual (Fig. 3c), with diverse and host-specific combinations of iSNVs across individuals, supporting the idea that viral genetic diversity is generated through independent within-host evolutionary processes and may accumulate more efficiently as the number of infected hosts increases.

Using NB regression for iSNVs identified in vaccinated groups after excluding variants detected in the positive control group, we observed a significant statistical increase in iSNV counts with increasing vaccine dose in the Spike, ORF1a, ORF1b and ORF3a genes, while other genomic regions showed no significant dose-associated effects (Fig. 4a). In contrast, when all iSNVs were included without excluding positive control-derived variants, the dose-associated effect remained significant in the Spike, ORF1a and ORF3a genes but not in ORF1b. Selection pressure analysis based on filtered dataset revealed that positive selection was observed in the Spike, ORF1b and ORF10 proteins, primarily driven by the 1 µg vaccine group, whereas lower dose groups showed no clear evidence of positive selection (Fig. 4b). Recent genome-wide analyses have shown that SARS-CoV-2 evolution is shaped by extensive epistatic interactions across the genome, with the Spike protein representing a major hub within this interaction network [34]. Population-level analyses have shown that mutations in the Spike protein increased following the introduction of vaccination, suggesting a shift in evolutionary pressure toward immune-relevant regions [35]. These findings suggest that mutations in the Spike protein may be associated with concurrent increases in mutations across other genomic regions, such as ORF1a, ORF1b and ORF10, indicating coordinated genome-wide evolutionary dynamics rather than independent gene-specific changes. In human patients infected with the Delta variant, vaccination was associated with increased within-host diversity at the whole-genome level; however, no evidence of increased selection pressure was observed [36]. Notably, these analyses primarily focused on genome-wide metrics without detailed characterization of non-spike proteins. Therefore, further investigation is required to elucidate whether coordinated mutational dynamics across non-spike genomic regions contribute to within-host viral evolution.

It is important to note that the dose-dependent increase in iSNV density was not restricted to NS mutations. Synonymous iSNVs also increased with vaccine dose in several genes, and in some genes the effect size for synonymous mutations was comparable to that observed for NS mutations. This suggests that the accumulation of iSNVs at higher vaccine doses may not be explained by selection alone. Reduced viral replication under stronger vaccine-induced pressure may decrease the effective viral population size, thereby increasing stochastic variation in allele frequencies and allowing both synonymous and NS variants to reach the 5% detection threshold more frequently. Therefore, the increase in NS iSNVs at higher vaccine doses likely reflects both selective pressure, as supported by the *pN/pS* analysis and underlying population-dynamic effects such as bottlenecking or genetic drift.

Compared with the first animal experiment, fewer iSNVs were observed in the second animal experiment. This reduction largely reflects the absence of detectable viral replication in the higher vaccination dose groups in the second experiment, which were, therefore, excluded from iSNV analysis, whereas in the 0.01 µg group, both viral titres and iSNV levels were comparable between the first and second experiments (Figs 2c, 3a and b). In the first animal experiment, higher vaccination doses were associated with reduced viral replication but increased iSNV diversity, suggesting that mutant generation is not directly proportional to viral replication and may instead be influenced by the level of vaccine-induced immunity. Furthermore, the second animal experiment likely involved a lower level of infectious viral inoculum compared with the first, as infectious virus was not detectable by plaque assay. This is consistent with previous studies suggesting that the initial inoculum may influence the likelihood of breakthrough infection, with higher viral inoculum potentially associated with reduced vaccine-induced protection and an increased probability of breakthrough infection [37]. Together, these observations suggest that the initial inoculum may influence the likelihood of successful breakthrough infection, while vaccine dose-dependent effects contribute to the generation of mutants and iSNV diversity. Therefore, iSNV diversity appears to be more closely associated with early infection dynamics and selective constraints, although contributions from viral replication cannot be excluded.

The majority of iSNVs identified in this study have also been observed as aa substitutions in SARS-CoV-2 sequences derived from human patients (Fig. 5 and Tables S4–S6). Interestingly, key aa substitutions characteristic of the Omicron variant were observed at epitope sites identified in two or more individuals within the vaccinated group, including N440K, H655Y, N679K, N764K, D796Y, Q954H and N969K (Fig. 5b), which have been widely reported as defining mutations of the Omicron variant [38–40]. The impact of specific aa substitutions can vary depending on the viral genetic background, called as an epistatic shift [41]. To assess the potential impact of individual-specific combinations of iSNVs on antibody binding, docking affinities between individual-specific viral variants and three representative SARS-CoV-2 neutralizing antibodies were evaluated [5]. *In silico* analyses incorporating individual-specific iSNVs revealed reduced binding affinity of class 1 and class 3 neutralizing antibodies (PDB ID: 7C01 and 6XDG) in a subset of individuals from the 1 µg group in the first trial, as well as in some individuals from the vaccinated groups in the second trial (Fig. 6a), indicating inter-individual variability. In contrast, binding affinity to the ACE2 receptor remained largely unaffected (Fig. 6). Stochasticity exists in viral transmission, and only a randomly selected subset of virions within the donor can contribute to superspreading [6]. Based on the findings of this study, certain combinations of iSNVs that emerged within individual hosts following vaccination are presumed to have an increased likelihood of contributing to superspreading events, potentially through epistatic shifts that enhance escape from pre-existing antibodies while preserving binding affinity to the ACE2 receptor (Fig. 6) [6]. Additionally, these findings suggest that preclinical analyses may provide partial insights into aa substitutions potentially associated with vaccine-induced immune pressure.

This study has several limitations. First, the recombinant spike protein vaccine used in this study represents a specific vaccine platform and may elicit immune responses distinct from those induced by other platforms, such as mRNA or viral vector vaccines. Therefore, the observed evolutionary dynamics and mutation patterns may not be directly generalizable to other vaccine platforms. Second, detailed characterization of cellular and humoral immune responses was not performed, limiting our ability to directly correlate immune response profiles with viral evolutionary dynamics. Further studies incorporating comprehensive immunological analyses across multiple vaccine platforms are needed to better understand the relationship between vaccine-induced immunity and viral evolution. In conclusion, this study provides experimental evidence that vaccine dose may be associated with viral genetic diversity and evolutionary trajectories during infection. Distinct viral replication patterns were observed between the URT and LRT, with higher vaccine dose groups showing more pronounced iSNV counts and signals of selection in the URT, particularly in the spike protein and selected non-spike viral proteins. Collectively, these findings suggest that vaccine-induced immune pressure may influence SARS-CoV-2 intra-host evolutionary dynamics and provide insights into the relationship between vaccine-induced immunity and viral evolution. Furthermore, findings from this preclinical animal model may provide limited insights into the potential prediction of vaccine escape-associated aa substitutions, although further validation is required.

This study also has limitations, including the relatively sparse number of detected iSNVs and the small number of animals in each experimental group, which may limit the statistical power and generalizability of the findings. In addition, because this study evaluated a recombinant spike-protein vaccine in a hamster challenge model, the observed evolutionary patterns should be interpreted within this specific experimental context and should not be over-generalized to other vaccine platforms, host species or natural infection settings.

## Supplementary material

10.1099/jgv.0.002314 Fig. S1.

10.1099/jgv.0.002314 Supplementary Material 1.

## Abbreviations

ACE2: angiotensin-converting enzyme 2
dpi: days post-infection
GISAID: Global Initiative on Sharing All Influenza Data
HADDOCK: High Ambiguity Driven protein-protein
IFN-γ: interferon gamma
IL-6: interleukin-6
IL-1o: interleukin-10
IP-10: interferon gamma-induced protein 10
IRR: incidence rate ratio
iSNV: intra-host single-nucleotide variants
LRT: lower respiratory tract
MIP-1: macrophage inflammatory protein 1
NB: negative binomial
NS: non-synonymous
NTD: N-terminal domain
qRT-PCR: quantitative reverse transcription polymerase chain reaction
RBD: receptor-binding domain
SARS-CoV-2: severe acute respiratory syndrome coronavirus 2
SRA: Sequence Read Archive
TNF-α: tumour necrosis factor alpha
URT: upper respiratory tract
VOC: variants of concern
