# Supplementary material for: Dose-dependent effects of recombinant spike protein-based vaccination on SARS-CoV-2 evolutionary dynamics and the emergence of variants in hamster models
Source: J Gen Virol. 2026 Aug 19;107(8):002314. doi: 10.1099/jgv.0.002314 (PMC13489668; doi:10.1099/jgv.0.002314)
Supplement: Supplementary file 1 [file jgv-107-02314-s001.pdf]

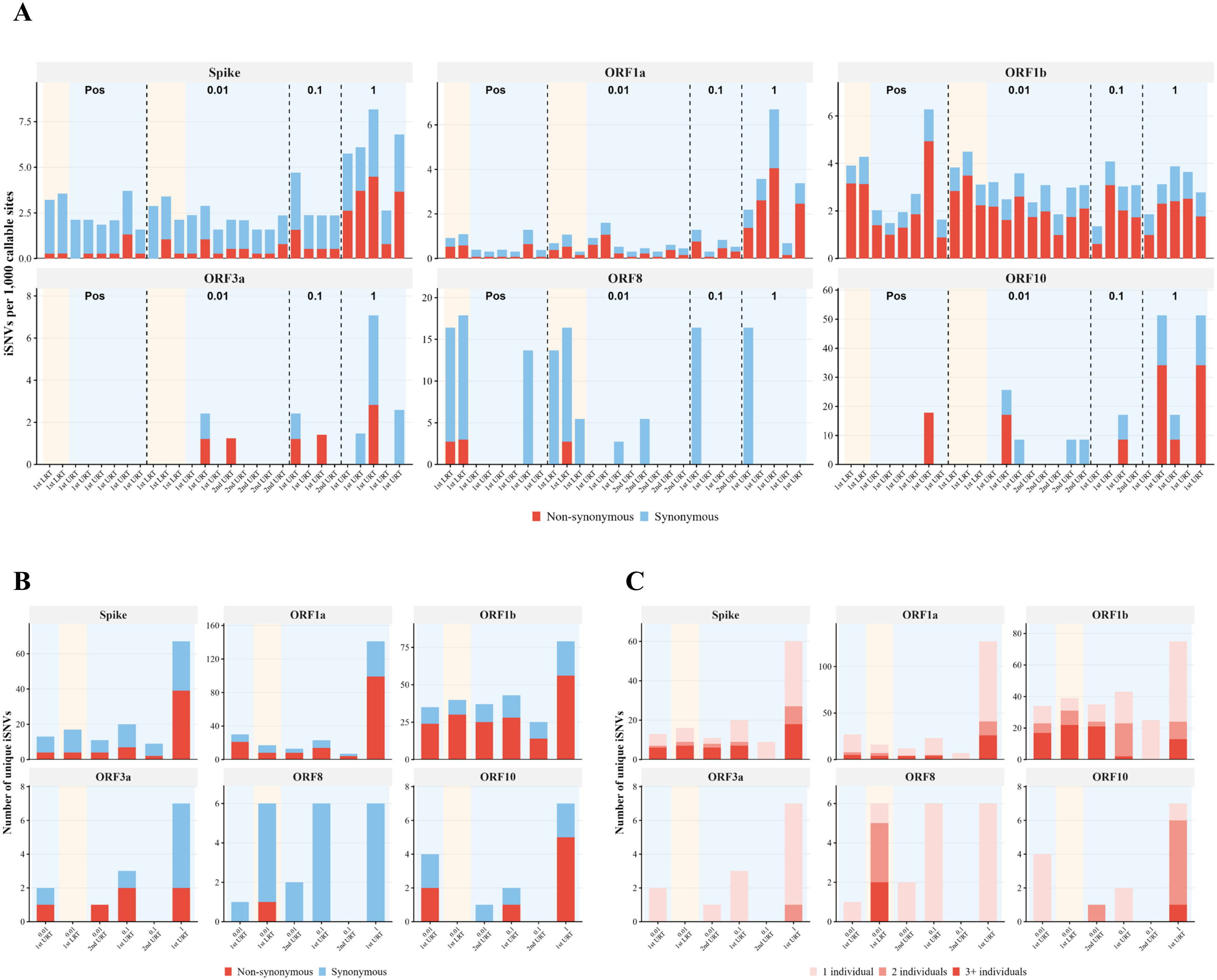

Supplementary Figure 1. Vaccine dose-dependent gene-specific iSNV density and inter-individual sharing, stratified by mutation type, including iSNVs detected in positive control groups.
